# EXPLORING QUALITY AND COVID MEASURES OF NEW YORK STATE LONG-TERM CARE FACILITIES INVOLVED IN THE SAFE STAFFING LAWSUIT

**DOI:** 10.1093/geroni/igac059.995

**Published:** 2022-12-20

**Authors:** Jason Semprini, Brian Kaskie

**Affiliations:** University of Iowa College of Public Health, Iowa City, Iowa, United States; University of Iowa, Iowa City, Iowa, United States

## Abstract

In April 2021, New York's "Safe Staffing" law capped Long-Term Care Facility (LTCF) profits. LTCFs with "excess profits" are now challenging the law in court. This study examined how LTCFs involved in the lawsuit differed from other NY state LTCFs before and during the COVID-19 pandemic. LTCF “Safe Staffing” lawsuit data were obtained from Long Term Care Community Coalition, then linked with Centers for Medicare and Medicaid Services COVID-19 and Small Business Association Paycheck Protection Program (PPP) data. First, we tested for differences across quality measures. We found that, compared to LTCFs not involved in the lawsuit, LTCFs in the lawsuit were more likely to be located outside of a hospital, report more certified beds and higher occupancy rates, and have higher overall quality scores. LTCFs in the lawsuit also reported lower staff ratings and staffing hours, which have previously been identified as a determinant of higher mortality in LTCFs. To create valid comparisons given these systematic differences, we specified “Doubly Robust” Augmented Inverse Probability Weighted regression models and tested if lawsuit involvement was associated with COVID-19 outcomes. Despite finding higher rates of admitting patients infected with COVID-19 in “excess profit” LTCFs, we did not find that COVID-19 deaths differed by lawsuit involvement. Finally, lawsuit involvement was associated with a higher probability of receiving a PPP loan. Before and during the pandemic, LTCFs with "excess profits" appeared fundamentally different than other NY LTCFs. How these differences impact the health of older adults receiving long-term care beyond the pandemic remains unknown.

